# Supplementary material for: Data-driven identification of total RNA expression genes for estimation of RNA abundance in heterogeneous cell types highlighted in brain tissue
Source: Genome Biol. 2023 Oct 16;24:233. doi: 10.1186/s13059-023-03066-w (PMC10578035; doi:10.1186/s13059-023-03066-w)
Supplement: Supplementary file 2 — Additional file 2. This additional file contains all supplementary figures and their captions. [file 13059_2023_3066_MOESM2_ESM.pdf]

Supplementary Material

Supplementary Figures

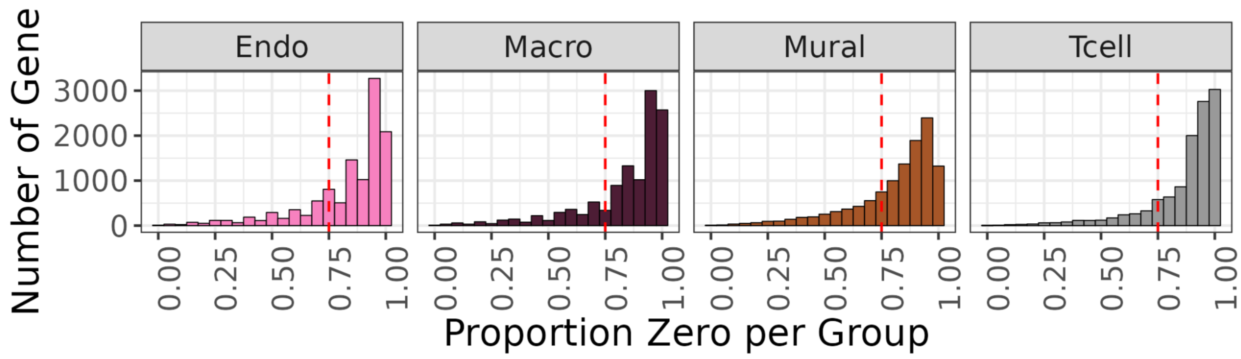

Fig S1: Distribution of Proportion Zero for rare cell types. Endothelial cell (Endo), Macrophage (Macro), Mural cell (Mural), and T-cell are cell types that have 100 (0.14%) or fewer nuclei in the snRNA-seq dataset used in this study (**Additional file 1: Table S1**). Given they are present in at most three of five brain regions, we did not include brain regions when grouping nuclei for the Proportion Zero filter calculation. Related to **Figure 2a**, Methods: *Expression and Proportion Zero Filtering*.

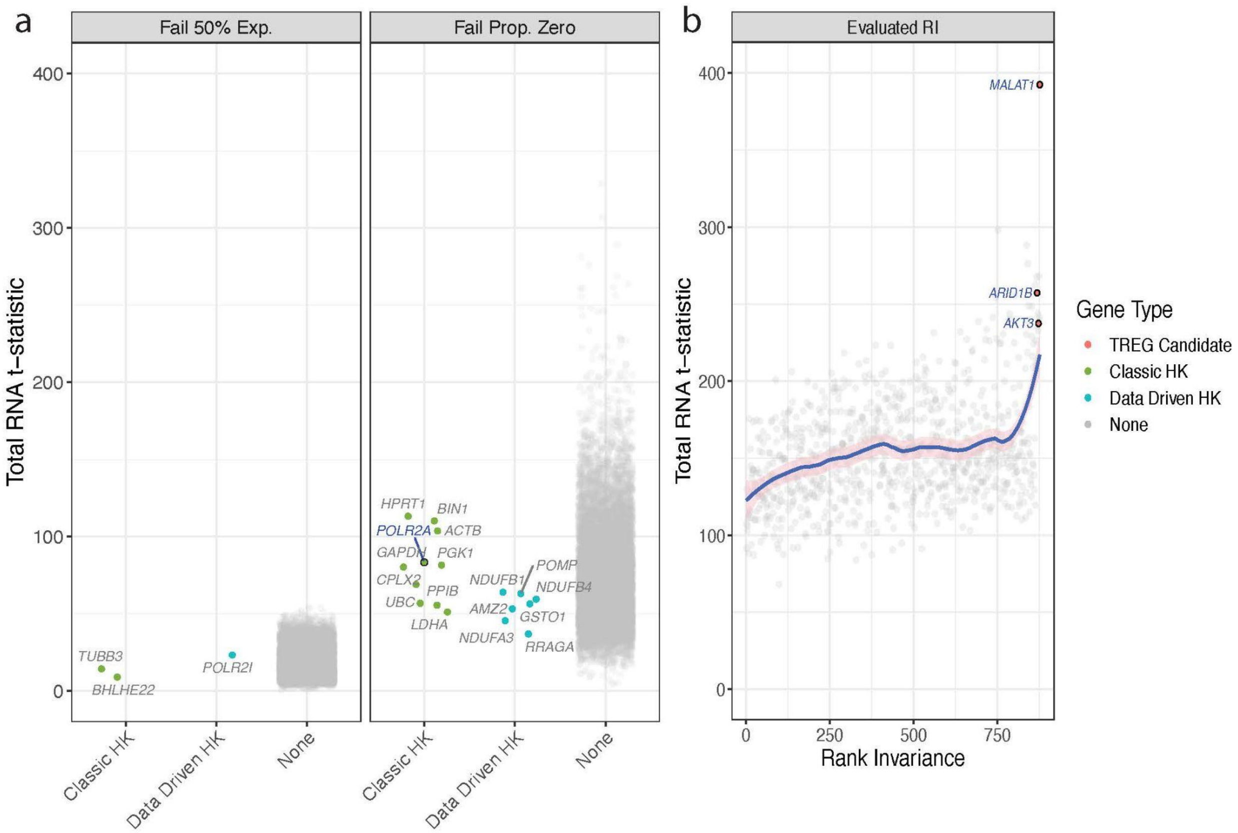

Fig S2: Relationship between Gene Properties and total RNA *t*-statistics. *t*-statistics are calculated for the relationship between total RNA expression (explanatory variable) and gene expression (response variable), adjusted for cell type (Methods: Total RNA linear regression) for all 23,038 genes in the reference snRNA-seq data set. **a.** The distribution of total RNA *t*-statistics for genes that failed either the top 50% filter (left panel) or the Proportion Zero filtering process (right panel). The genes are annotated by color for classic housekeeping (HK) genes [46, 47], brain data-driven housekeeping (HK) genes [6], or belonging to none of those three groups. **b.** Scatterplot of the relationship between the Rank Invariance and the total RNA *t*-statistic for the 877 genes that pass all the filtering steps. Candidate TREGs explored with RNAscope are highlighted in red and labeled. A loess regression line with span = 0.3 is shown. All three panels are mutually exclusive categories of genes based on the filtering processes. Related to **Figure 2b** and **Figure 3c-d**.

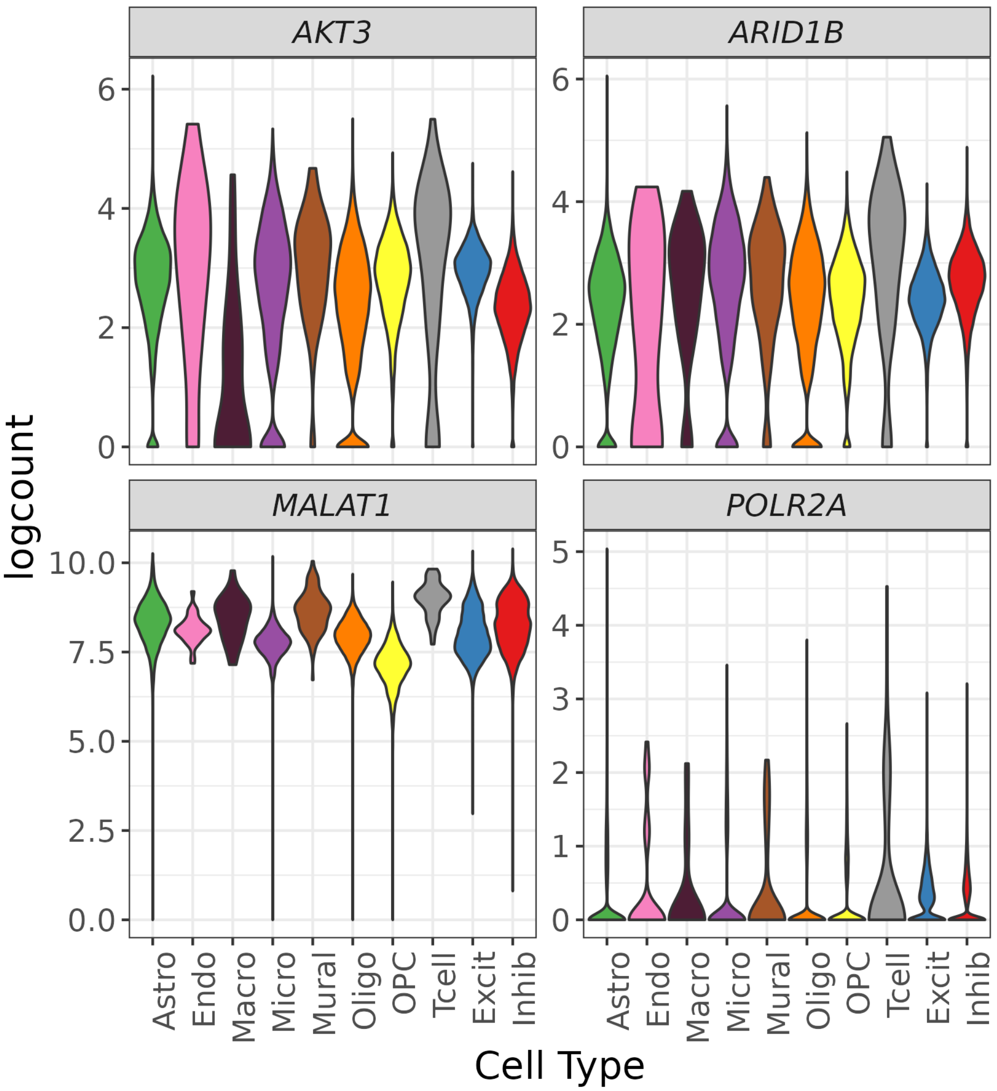

Fig S3: snRNA-seq expression plots. Distribution of the normalized log2-transformed expression over cell types for *AKT3*, *ARID1B*, *MALAT1*, and *POLR2A*. Candidate TREGs show less expression variability across most cell types compared to *POLR2A*. Expression Ranks (**Figure 3b**) are less variable than measured expression values since the Expression Rank takes into consideration the context of the expression levels of other genes in a given cell.

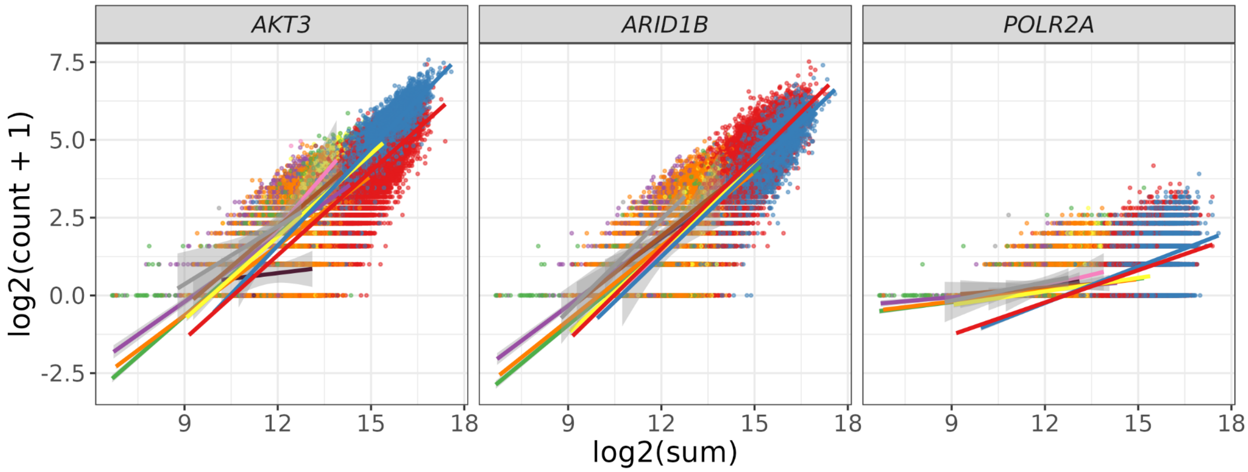

Fig S4: **Relationship between total nuclear expression and expression of candidate TREGs**. Scatter plot of the total RNA expression (estimated by the nuclei log2 sum of all counts) against the nuclei gene expression (log2 of the count plus one), and *AKT3*, *ARID1B, and POLR2A*; related to **Figure 3d**. The scatter plots are overlaid with the linear fit for each cell type and colored by cell type. See **Figure 3c** for *MALAT1*.

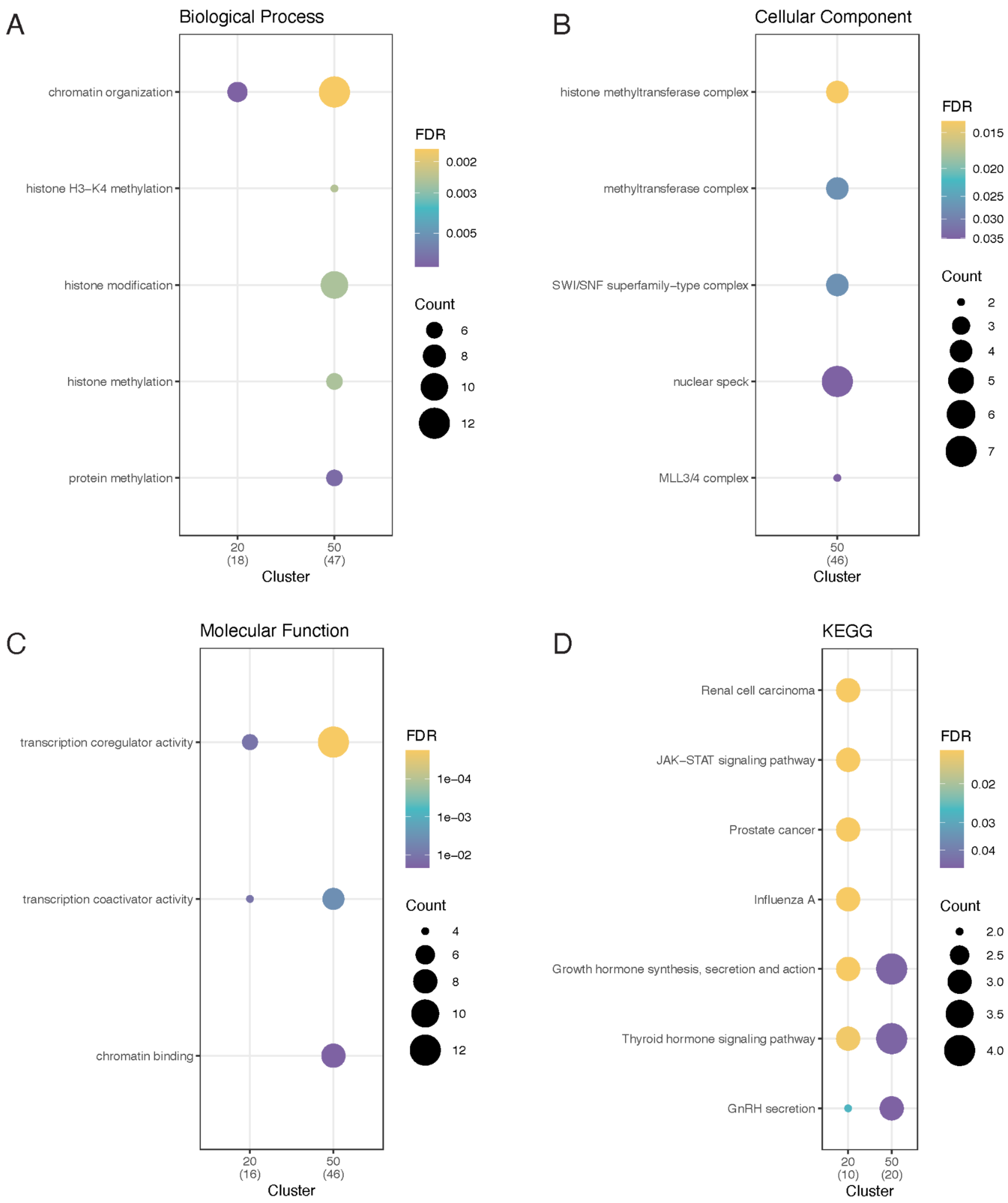

Fig S5: Gene ontology and KEGG dotplots. Gene ontology enrichment results for the top 20 or top 50 candidate TREGs found using snRNA-seq data from 5 human brain regions across ontologies for (**A**) biological processes (BP), (**B**) cellular components (CC), (**C**) molecular function (MF), or (**D**) KEGG pathways. The top candidate TREGs are enriched for functions related to chromatin organization, transcription co-regulator/activator activity, namely RNA

splicing and histone modification, as well as related cellular components and biological processes. This finding is consistent with the TREG property that its expression is associated with transcriptional activity. Related to **Figure 3c-d**.

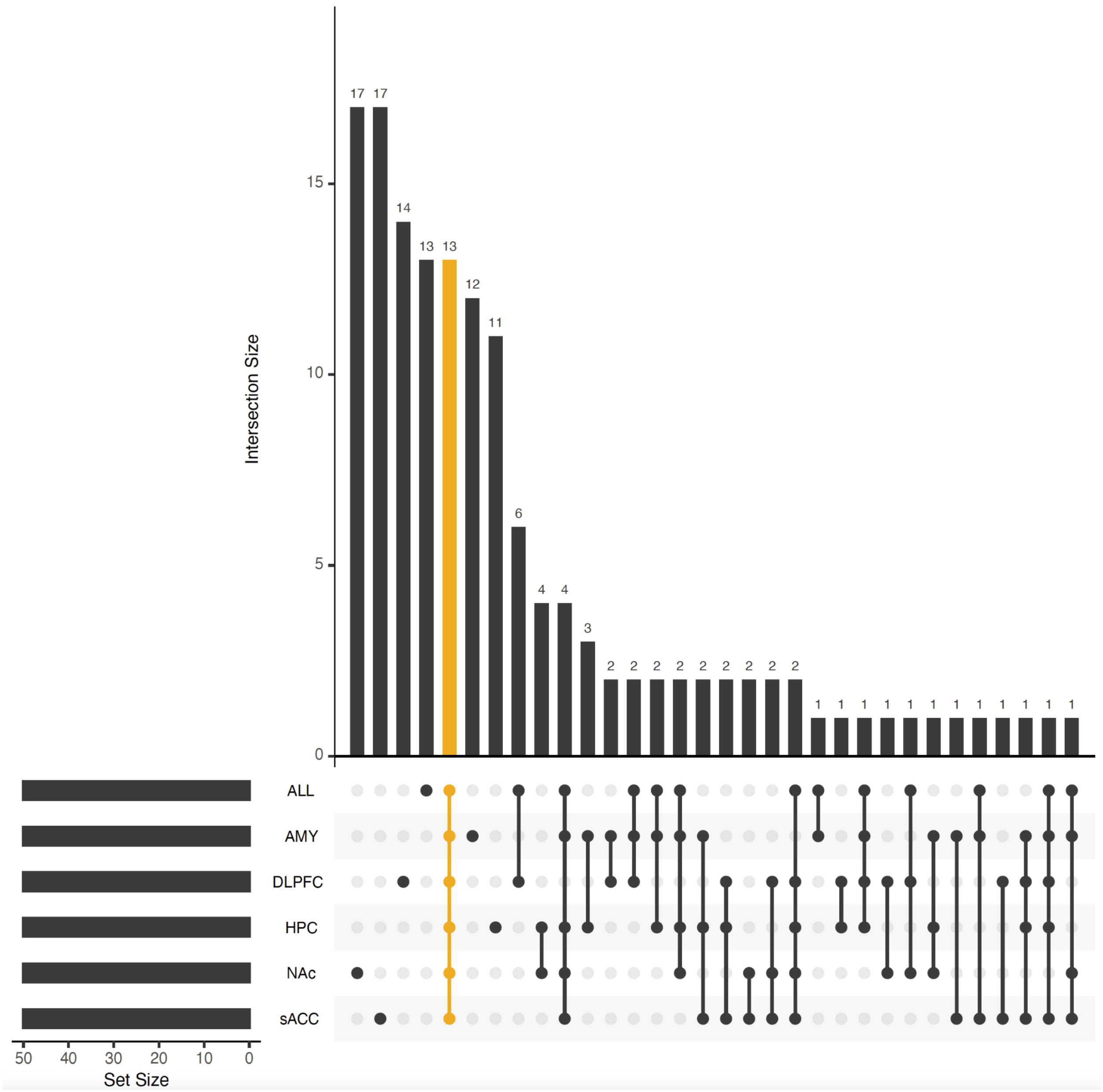

Fig S6: Upset plot for top 50 RI genes. The Proportion Zero filtering and Rank Invariance calculation was performed on each of five brain regions and across all brain regions. The overlap of the sets of the top 50 Rank Invariance genes from each analysis is shown in this "UpSetR" plot [45]. An "UpSetR" plot is similar to a Venn diagram as it shows bars denoting the intersection size (top) for different types of intersections (bottom right) based on sets of features (in this case gene IDs) that could have different sizes (bottom left, in this case all six sets are of equal size: 50 genes). "UpSetR" plots can include more sets than Venn diagrams. The intersections are ordered by decreasing intersection size, and here the fifth largest intersection corresponds to the intersection across all six sets (shown in orange). The genes *AKT3*, *ARID1B*, and *MALAT1* are part of the set of 13 genes that are present in each set of top 50 Rank Invariance genes (**Additional file 1: Table S3**).

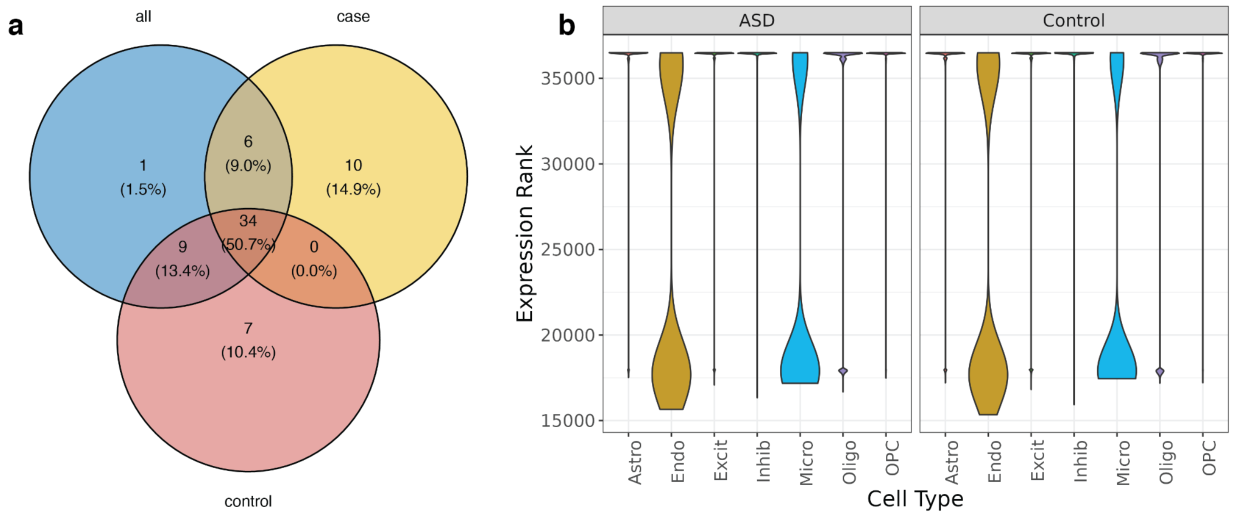

Fig S7: **Stability of TREG Identification in a Case-Control Dataset. a.** Venn diagram of top 50 candidate TREGs from Velemshev et al. data evaluated by all samples, or subset to only case or control samples. Percent values were calculated from the intersected set of 67 unique genes found between the three analyses. **b.** The distribution of the Expression Ranks (*y*-axis) over all cell types (*x*-axis) for the example TREG candidate *CADM2* shows Expression Rank stability across most cell types and between diagnosis.

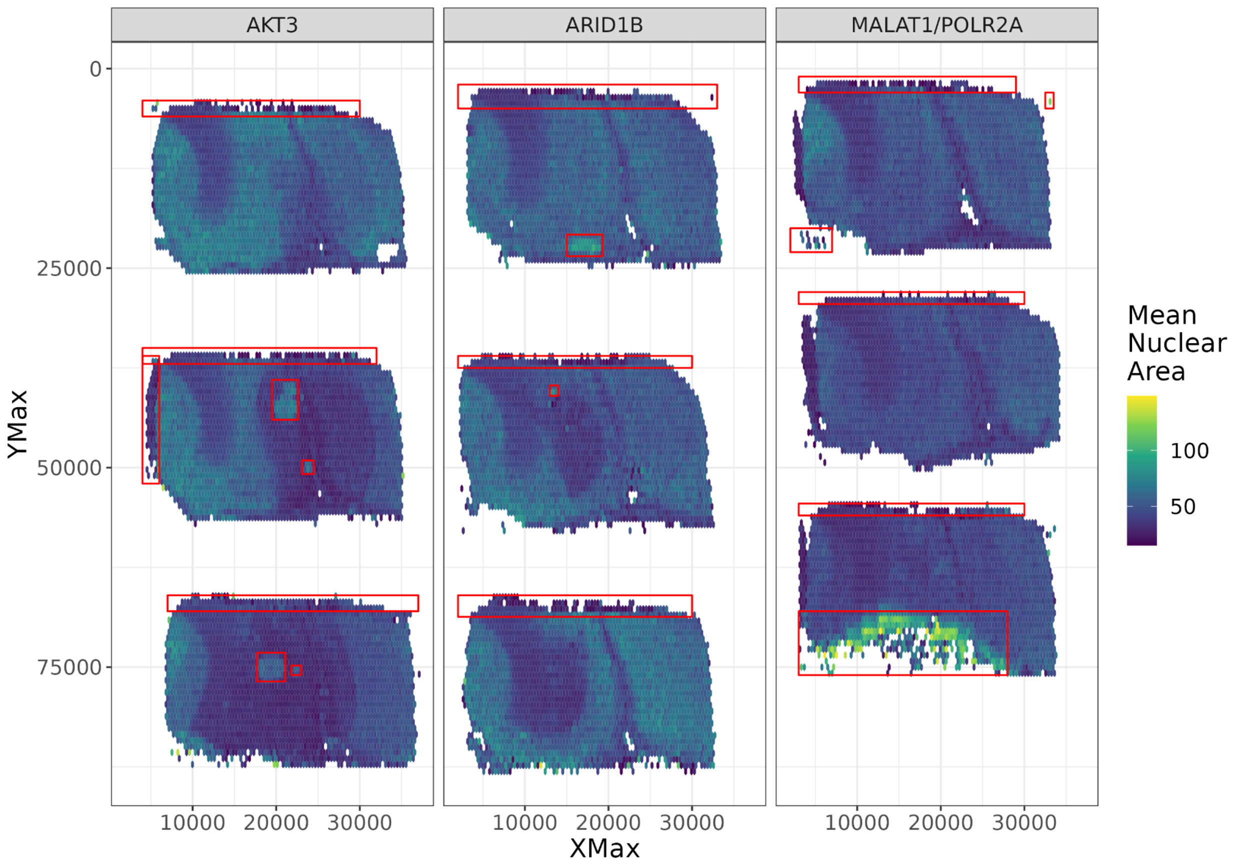

Fig S8: Spatial distribution of mean nuclear area across all *n* = 9 tissue sections. To visualize the nuclear area spatially, we used hex bins to compute the mean nuclear area per hex bin. The regions inside of the red boxes were flagged during quality control for unusually high nuclear size, and the enclosed nuclei were excluded from the analysis for technical artifacts due to sampling and imaging. Related to **Figure 6a**, Methods: Quality control and spatial quantification of HALO data.

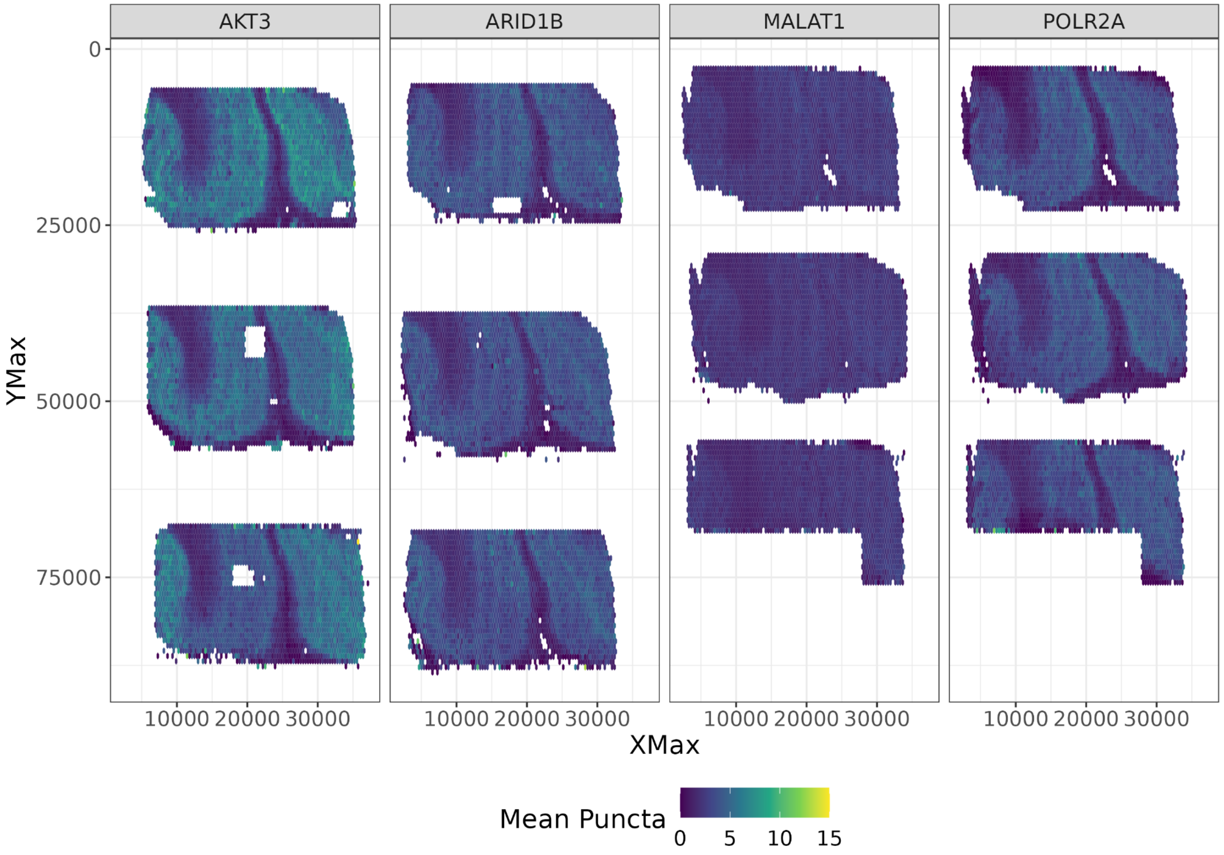

Fig S9: Spatial distribution of mean number of puncta across all *n* = 9 tissue sections, post quality control. Similar to **Additional file 2: Fig S8**, but for the mean number of puncta for each gene after performing quality control. Given that *MALAT1* and *POLR2A* were multiplexed in the same experiment on a single slide, (**Figure 4a**, **Additional file 1: Table S4**), their spatial shapes are identical. Related to **Figure 6b.**

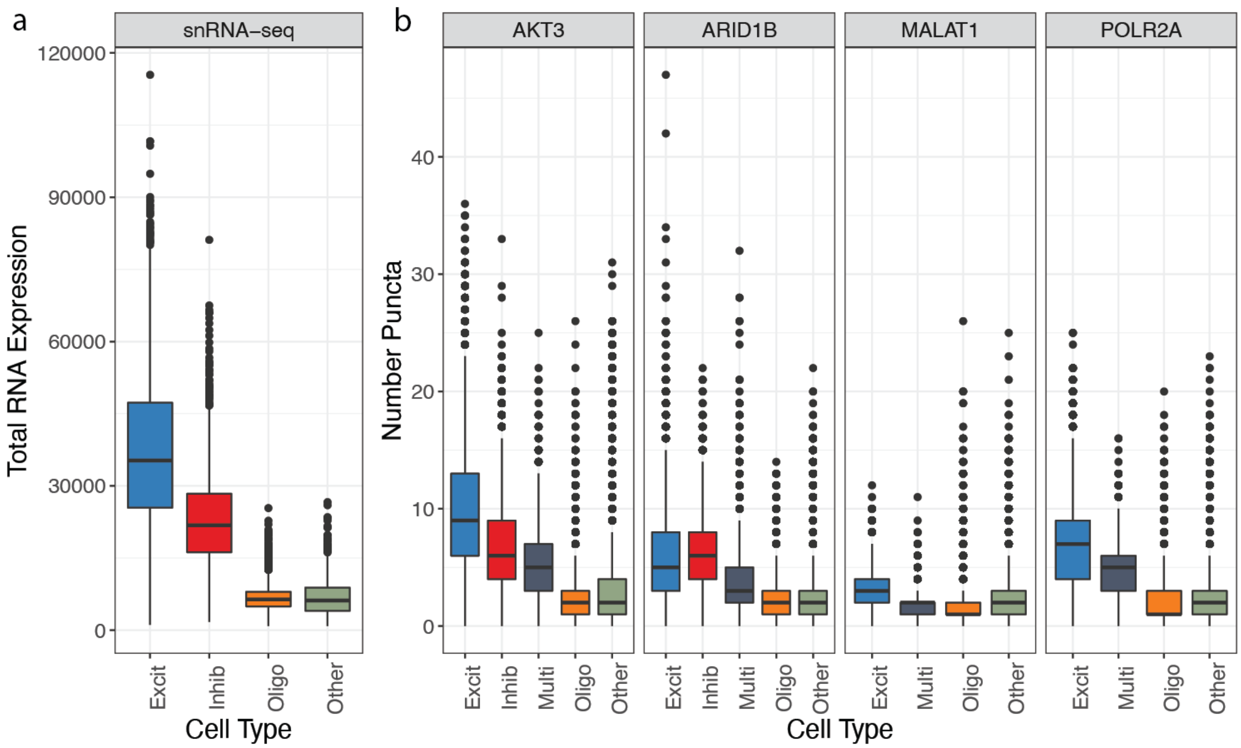

Fig S10: Boxplots of total nuclear RNA expression across all cell types. **a.** Total nuclear RNA expression summed from snRNA-seq data. Nuclei not classified as Excit, Inhib, or Oligo were classified as “Other" and likely represent other non-neuronal cell types located in the brain, such as astrocytes and microglia. **b.** Total nuclear RNA expression estimated by the number of puncta measured by RNAscope for each observed gene. Nuclei expressing more than one cell type marker (Excit, Inhib, or Oligo) are classified as “Multi” as they cannot be definitively assigned a cell type phenotype due to overlapping fluorescent signals. Related to **Figure 7** and **Table 1**.

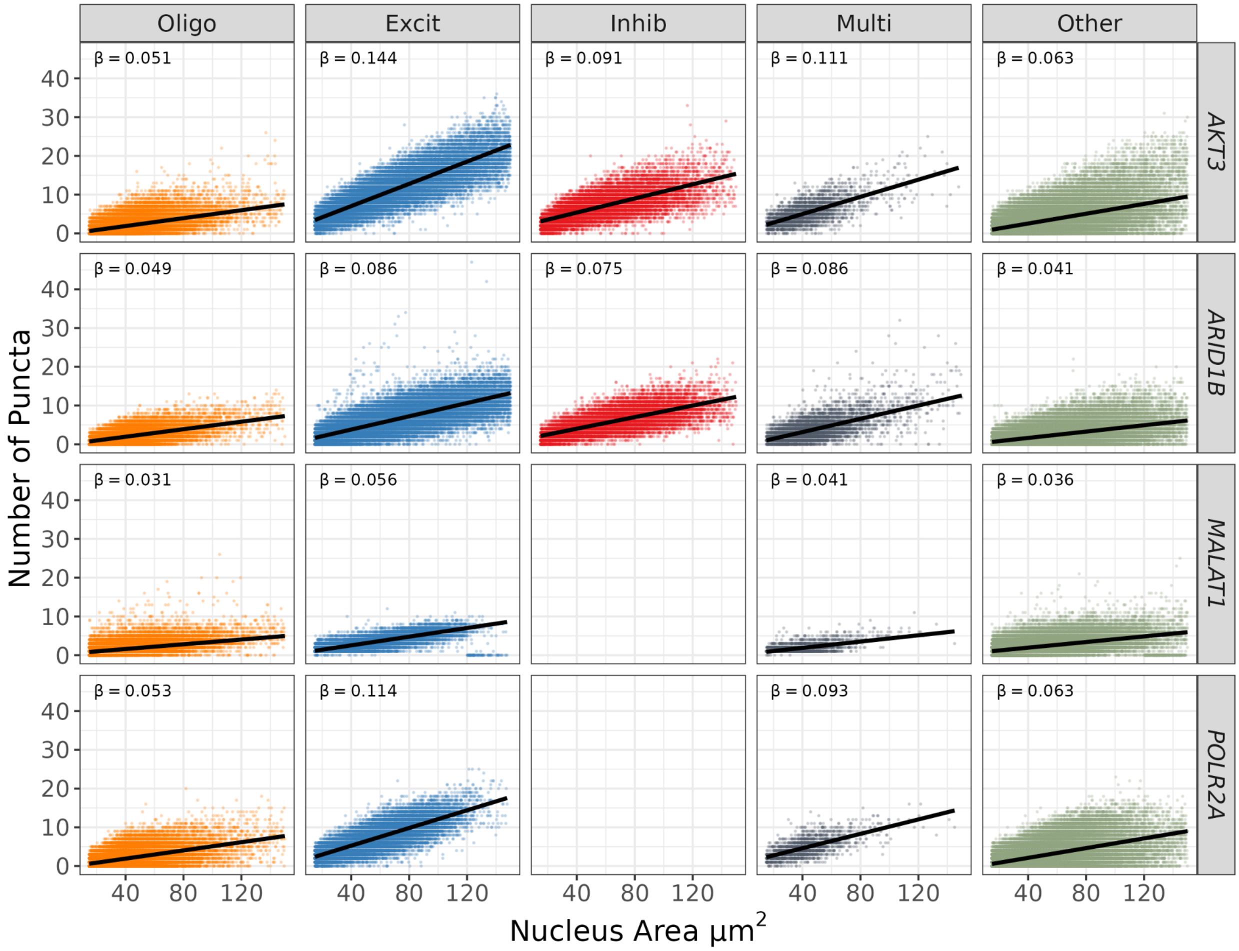

Fig S11: Nuclear puncta versus nuclear area across cell types. Given the paired nuclear size (area in μm^2), RNA abundance (number of puncta), and cell type assignment data from RNAscope experiments, we can examine the relationship between puncta and area across all cell types. The relationship between nuclear area and puncta is different across cell types, for example excitatory neurons (Excit) and oligodendrocytes (Oligo) for *AKT3* have a difference in slopes of 0.093 ($t=155.77$, $p<2*10^{-16}$).Cell types are the same as those from **Additional file 2: Fig S10** and it should be noted that *MALAT1* puncta were unreliable due to oversaturation of fluorescent signals as previously explained. Related to **Figure 7**. There is no data for inhibitory neurons for *MALAT1* and *POLR2A* as *GAD1* was omitted for this experiment due to limitations in multiplexing (**Figure 4**).
