# Supplementary material for: Data-driven identification of total RNA expression genes for estimation of RNA abundance in heterogeneous cell types highlighted in brain tissue
Source: Genome Biol. 2023 Oct 16;24:233. doi: 10.1186/s13059-023-03066-w (PMC10578035; doi:10.1186/s13059-023-03066-w)
Supplement: Supplementary file 3 — Additional file 3. This additional file contains the review history. [file 13059_2023_3066_MOESM3_ESM.docx]

**1^st^ round**

**Reviewer 1**

Huuki-Myers et al. presented a new software for identifying a new class of "control" genes called Total RNA Expression Genes (TREGs), which finds genes that are correlated with total RNA abundance in heterogeneous cell types. Their software is easy to install and works well on their sample dataset. Their paper identified a new set of genes, TREGs that have never been studied. I have a few concerns with the software. Please see my comments below.

1. This is a conceptual question. TREGs are defined as a set of genes consistently highly expressed across cell types with some variation. The consistency is measured by the correlation between their expression with total RNA expression. Reading through the article, I do not feel strong enough that we should use TREGs rather than total RNA expression when they are also available. In the Background section, you provided an example of deconvolution, where total RNA abundance is crucial for cell type proportion estimation. This is an excellent example of showing the importance of total RNA abundance. Can you provide some examples where TREGs cannot be replaced by total RNA abundance or total RNA abundance are not available?

2. In the deconvolution framework, there are two types of "cell-type proportions": (1) cell number proportions, which is an intuitive definition estimated by reference 12. (2) molecule proportions from each cell type. Nowadays, more and more methods developed with this definition of cell-type proportions. The difference between the two definitions is the total RNA abundance. I suggest that you make it more explicit about it.

3. In this article, Huuki-Myers et al. only considered brain tissue. I suggest adding examples for TREGs using different tissues or changing the title by restricting their analysis to brain tissue.

4. The authors provided a good discussion of TREGs and housekeeping genes. It will be better to see how TREGs result when applying to data of different conditions, say disease(case)/control.

5. In the procedure of selecting TREGs, is the cut-off pre-determined? Please discuss more on how the select reasonable cut-offs when choosing TREGs.


Minor comments:
1. Please add figure legends to Figure 3.

**Reviewer 2**

Advances in next-generation sequencing technologies facilitate the exploitation of expression of genes from various perspectives, i.e., stable expression, cell-type specific expression, and spatially variable expression. In this study, the authors proposed a statistic model to identify Total RNA Expression Genes (TREGs), which are highly related to the total RNA abundance in heterogeneous cell types, and transcriptional activity. By applying the proposed algorithm to the human brain, the authors identify the critical genes of brains.

In all, the topic is interesting, and it will receive great interesting. Further However, there are also many problems:

1. On the algorithm issue, TREGs are identified with rank-invariance to identify stably expressed genes, where these genes that are consistently ranked are extracted. There are three factors invovled, i.e., criteria for removing lowly expressed genes, ranking strategy for genes, and statistic to to measure consistence. Among these three issues, the authors select 50% of genes for TREGS, what is the rationale for the threshold 50%? Furthermore, scRNA-seq data is extra-ordinary sparse, where most of genes are unexpressed. Why do not you remove all these unexpressed genes? There is also thresholdd for remove genes with maximum proportion zero (75%). Second, the consistence rank largely depends on the number of cell types, and statistic. However, the authors ignore these two issues. Therefore, the robustness of the proposed method also needs to be addressed.

2. On the identified TREGs issue, the authors are suggested to exploit the difference between TREGs and non-TREGs from biological perspectives, such as structure, function, etc. Identifying house-keeping genes are insufficient to fully validate the performance of the proposed methods.

3. As methodology, more data are needed to fully validate the performance of the proposed methods.

4. Finally, as the authors assert that TREGs are highly related to transcriptional activity. Thus, is it possible to generate transcription regulation model with TREGs?

**Authors’ response**

Reviewer #1

**Huuki-Myers et al. presented a new software for identifying a new class of "control" genes called Total RNA Expression Genes (TREGs), which finds genes that are correlated with total RNA abundance in heterogeneous cell types. Their software is easy to install and works well on their sample dataset. Their paper identified a new set of genes, TREGs that have never been studied. I have a few concerns with the software. Please see my comments below.**

We thank the reviewer for their time and interest in this work, and for the positive feedback on the TREG software.

**1. This is a conceptual question. TREGs are defined as a set of genes consistently highly expressed across cell types with some variation. The consistency is measured by the correlation between their expression with total RNA expression. Reading through the article, I do not feel strong enough that we should use TREGs rather than total RNA expression when they are also available. In the Background section, you provided an example of deconvolution, where total RNA abundance is crucial for cell type proportion estimation. This is an excellent example of showing the importance of total RNA abundance. Can you provide some examples where TREGs cannot be replaced by total RNA abundance or total RNA abundance are not available?**

We agree with the reviewer that in cases where the whole transcriptome is quantified, there is no reason to use an approximate measurement for total RNA expression when you can directly measure the real value, such as in single cell or single nucleus (sc/sn)RNA-seq. However, we envision TREGs to be used for approximating total RNA expression specifically in experimental assays that are limited to a small subset of the full transcriptome, but can add other important features to the data, such as cell size and shape.

The assay we highlight is single molecule fluorescent in situ hybridization (smFISH) RNA-scope, which can only measure a subset of genes (four in our setup), but offers other important cellular features, such as cell size, nuclear area, spatial position, and morphological features, that are not quantified in sc/snRNA-seq. Connecting these features, specifically nuclear area (**Supplementary Figure 11**), to total RNA expression as estimated by TREG expression may be important for downstream computational analyses, such as deconvolution.

To make this point more clear to readers we have added a sentence to the abstract:

“TREGs allow estimation of the total amount of RNA when an experimental assay is restricted to quantifying a limited number of genes.”

We have also clarified our motivation for the application of TREGs by revising several paragraphs in the background.

In the discussion of our manuscript, we added further examples other assays that are not transcriptome-wide and quantify only a limited number of experimenter-selected genes that also may have some application of TREGS.

“While our list of candidate TREGs could be valid for other DLPFC studies (**Supplementary Table 3**), we recommend that you use our R package with your own sc/snRNA-seq data. TREGs could be useful for other research purposes and other contexts than the ones envisioned here. **For example, emerging spatial transcriptomics technologies such as MERFISH and Xenium (where a limited number of probe targets must be selected), can employ TREGs as a complementary class of genes to traditional housekeeping genes to aid in cell type characterization.**”

We hope these additions clarify the application and utility of TREGs. The changes we made to address this reviewer’s comment are also applicable to the next comment.

**2. In the deconvolution framework, there are two types of "cell-type proportions": (1) cell number proportions, which is an intuitive definition estimated by reference 12. (2) molecule proportions from each cell type. Nowadays, more and more methods developed with this definition of cell-type proportions. The difference between the two definitions is the total RNA abundance. I suggest that you make it more explicit about it.**

We agree with the reviewer that “cell-type proportion” must be defined with detail. For readers of this manuscript, we have added a sentence to emphasize that this poses a challenge to deconvolution methods and cited our recent preprint that goes more in-depth to this discussion.

In this preprint we got into more detail about the definition of cell type proportion. In particular we say:

“**Cell type scale factor transformations can improve the performance of deconvolution algorithms.** While bulk transcriptomics deconvolution commonly predicts cell type proportions from expression data, it was noted that this approach may instead quantify total mRNA content in the absence of an adjustment for systematic differences in size and expression activity at the cell type level (3). This adjustment, which we will call a ‘cell type scale factor transformation’ (or cell scale factors for short), is used to transform the reference atlas (Z) data prior to deconvolution (3,41). It was introduced for microarray-based expression data (41,42) and later used for scRNA-seq data in multiple tissues (3,43,44). Cell scale factors are frequently used to generate

sc/snRNA-seq-based data that resemble real bulk RNA-seq data based on “pseudobulking” or aggregating molecular profiles across sc/snRNA-seq data (45). Reference atlas transformation using orthogonal and non-orthogonal cell scale factors reduced errors from deconvolution-based cell proportion predictions. This may be because estimates without this transformation quantify total RNA rather than cell proportion (3). Cell scale factors may be estimated from either expression or expression-orthogonal data, such as sorted or purified populations of immune cells, which are used in existing deconvolution algorithms such as *EPIC* and *ABIS* (43,44). The algorithms *MuSiC* and *MuSiC2* (16,18) can use either expression-based or user-defined scale factors (**Table 4**). Importantly, there are currently no standards for applying cell scale factors prior to deconvolution, and users may need to transform the reference atlas (Z) prior to calling certain algorithms. Further, many algorithms have not been extensively tested in complex tissues, such as brain, that show large differences in size and transcriptomic activity across cell types. Ultimately, more reliable cell scale factor estimation and standardized transformation procedures can facilitate future deconvolution research (3,41).”

Preprint cited: Maden, S. K., Kwon, S. H., Huuki-Myers, L. A., Collado-Torres, L., Hicks, S. C., & Maynard, K. R. (2023). [2305.06501] Challenges and opportunities to computationally deconvolve heterogeneous tissue with varying cell sizes using single cell RNA-sequencing datasets. *arXiv*. https://doi.org/10.48550/arXiv.2305.06501

Text added to background:

“With the exception of two methods [12, 13], the majority of existing bulk RNA-seq deconvolution methods [10] fail to incorporate this variation and hence report potentially biased estimates of the *relative* fraction of RNA attributable to each cell type rather than the true proportion of cell types [14]. **This is a challenge for bulk RNA-seq deconvolution algorithms that have to properly take into account heterogeneous cell types when estimating the proportions of cell types** [15]. However, methods to robustly estimate cell or nuclear size and total RNA abundance in the same assay are limited as approaches that capture global RNA expression, such as snRNA-seq, require tissue homogenization preventing the acquisition of cell size measurements. **Here we introduce Total RNA Expression Genes (TREGs), which can be used in combination with histological approaches such as single molecule fluorescence in situ hybridization (smFISH) to link estimates of cell size and total RNA abundance in the same assay.**”

**3. In this article, Huuki-Myers et al. only considered brain tissue. I suggest adding examples for TREGs using different tissues or changing the title by restricting their analysis to brain tissue.**

While we would be excited to validate this methodology in a range of tissues, we unfortunately are limited to smFISH validation experiments in brain tissue. While other sc/snRNA-seq

datasets are available to identify TREGS, we do not have access to those tissues to perform follow up smFISH experiments to validate our findings. A key component of our study is that we experimentally validated our computational findings, so we do not feel adding lists of TREGs from other tissues would significantly strengthen the study without validation by smFISH.

Based on the reviewers feedback we have edited the title as follows to make the scope of this paper more clear:

“Data-driven Identification of Total RNA Expression Genes (TREGs) for Estimation of RNA Abundance in Heterogeneous Cell Types **Highlighted in Brain Tissue**”

**4. The authors provided a good discussion of TREGs and housekeeping genes. It will be better to see how TREGs result when applying to data of different conditions, say disease(case)/control.**

The reviewer brings up an important concern, that TREGs be generalizable across conditions in a similar fashion as housekeeping genes are. To address this concern we have utilized the Velmeshev et al., autism spectrum disorder snRNA-seq dataset to generate sets of candidate TREGs within cases only, control only, and across all samples as an additional analysis in the paper evaluating the performance of our methodology across disease states.

We found that out of the top 50 genes, found throughout the rank invariance process, 34 overlapped all three sets of candidate TREGs (50.7% of the 67 unique identified genes). We also identified the gene *CADM2* which showed high rankings in all three evaluations of the Velmeshev et al. data and was our 10th ranked TREG in our data. This shows that there is stability between TREGs in different cases such as disease state, as well as some select genes being stable between datasets. Unfortunately it is out of the scope of this manuscript to validate these TREGs with a RNAscope experiment (again due to lack of tissue availability), but *CADM2* could be a strong candidate for future validation.

We added the following sections to the manuscript based on the reviewer’s feedback: Results:

*Identification of TREGs in a Case-Control Dataset*

To examine the stability of the method using data collected from a disease state and control donors, we performed the Rank Invariance workflow on a snRNA-seq dataset containing samples from neurotypical donors and donors with autism spectrum disorder from Velmeshev et al. [24]. We identified a list of the top 50 candidate TREGs from this dataset as a whole, then separately for the case and control samples. We found that 34 genes (50.7% of 67 unique genes between all sets) were present in all three sets of candidate TREGs (**Supplementary Figure 7a**). This shows that the Rank Invariance workflow is able to identify the same TREG candidates between disease states. *MALAT1* was the top Rank Invariance gene in all three conditions. We also noted the gene

*CADM2* was identified in the top 15 candidate TREGs across our evaluations of the Velmeshev et al. dataset, as well as in the top ten TREGs identified in our primary analysis in the Tran-Maynard et al. data. Like other highlighted TREG candidates, CADM2 shows Expression Rank stability across most cell types and between diagnosis (**Supplementary Figure 7b**).

Discussion:

The Rank Invariance method demonstrated stability over biological states by returning a similar set of candidate TREGs across disease states as evaluated in the Velmeshev et al. dataset [24]. The gene *CADM2* was a highly ranked TREG candidate when evaluating either case or control data separately, or the full dataset (which we recommend), as well as in the control only Tran, Maynard et al. dataset [20]. *CADM2* may be another TREG of interest for hu

.man cortex, which could be validated in future studies.

**Supplementary Figure 7**: **Stability of TREG Identification in a Case-Control Dataset. a.** Venn diagram of top 50 candidate TREGs from Velemshev et al. data evaluated by all samples, or subset to only case or control samples. Percent values were calculated from the intersected set of 67 unique genes found between the three analyses. **b.** The distribution of the Expression

Ranks (*y*-axis) over all cell types (*x*-axis) for the example TREG candidate *CADM2* shows Expression Rank stability across most cell types and between diagnosis.

Methods:

*External Data Replication in Case-Control Data*

A second snRNA-seq dataset from Velmeshev et al, case (autism spectrum disorder) and control samples from the prefrontal cortex and anterior cingulate cortex was used to validate this process in a more diverse biological context [24]. Cell types were evaluated at a similar seven broad cell type level as used in the Tran et al. data for the main

analysis. The data was evaluated in all samples, then case samples only, and control samples only. In the *Expression and Proportion Zero filtering* step, the Proportion Zero limit was set to 0.85 for all three groups instead of 0.75 as this dataset was more sparse than the Tran et al dataset. The *Rank Invariance calculation* was performed on the resulting set of genes for each group. The fifty genes with the highest Rank Invariance were considered TREG candidates, and compared to evaluate stability of the analysis between the case and control conditions.

**5. In the procedure of selecting TREGs, is the cut-off pre-determined? Please discuss more on how the select reasonable cut-offs when choosing TREGs.**

When identifying candidate TREGs with our software, there are a few thresholds users will select. In our manuscript, we used a few filters.

● We focused on genes among the top 50% of genes expressed in the snRNA-seq data. This helped address sparsity inherent to snRNA-seq data.

● We used a maximum Proportion Zero filter of 75%.

Overall it’s a balancing act between the computational requirements (reduce sparsity inherent to snRNA-seq data, reduce expression rank ties) and the biological goal (select a gene expressed in most nuclei from all cell types of interest). While we consider these to be thresholds that balance both aspects and are practical, ultimately we do encourage users to plot their own data. For example, making plots like those from **Supplementary Figure 2**. **Supplementary Figure 2A** is useful to examine whether genes you might have expected to pass the filters are being dropped. You can then check whether they were just below the filtering cutoffs, or significantly far away from them. Once you have the candidate TREG results, then **Supplementary Figure 2B** is useful to examine at what point do the top candidate TREGs have a stronger relationship with total RNA expression as measured from the snRNA-seq data. That is, where the blue curve jumps up and shows a more clear association between the two axes of that plot. Among the top candidate TREGs, there might be practical limitations to consider for using that TREG in another assay, such as availability of RNAscope probes as well as measurability of the puncta for a given probe. We showed in **Figure 5** how *MALAT1* could not be reliably quantified with RNAscope due to high expression and oversaturation of fluorescent signals. In the case of RNAscope, we recommend testing the measurability of candidate TREGs with RNAscope data before generating a full dataset with a probe that may be difficult to accurately quantify.

To address this reviewer’s concern, we have expanded the software documentation (see https://github.com/LieberInstitute/TREG/commit/e6f7f5bebc1e02dfeb5c6a90e28dd760e7025fcf) and added a section on selecting thresholds at

http://research.libd.org/TREG/articles/finding_Total_RNA_Expression_Genes.html#selecting-thr esholds.

**Minor comments:**

**1. Please add figure legends to Figure 3.**

We added the color legend for Fig 3. The colors representing cell types are consistent across all figures. Thanks for this comment, see the updated figure below.

Reviewer #2

**Advances in next-generation sequencing technologies facilitate the exploitation of expression of genes from various perspectives, i.e., stable expression, cell-type specific expression, and spatially variable expression. In this study, the authors proposed a statistic model to identify Total RNA Expression Genes (TREGs), which are highly related to the total RNA abundance in heterogeneous cell types, and transcriptional activity. By**

**applying the proposed algorithm to the human brain, the authors identify the critical genes of brains.**

**In all, the topic is interesting, and it will receive great interesting.**

Thank you for your time in revising our work. We agree with the reviewer that TREG will be of interest to a broad audience such as the readership of *Genome Biology*.

**Further However, there are also many problems:**

**1. On the algorithm issue, TREGs are identified with rank-invariance to identify stably expressed genes, where these genes that are consistently ranked are extracted. There are three factors invovled, i.e., criteria for removing lowly expressed genes, ranking strategy for genes, and statistic to to measure consistence. Among these three issues, the authors select 50% of genes for TREGS, what is the rationale for the threshold 50%?**

We were interested in identifying single genes that we can quantify in RNAscope and whose puncta measurements per cell are indicative of the total transcription of each cell (or nuclei). Biologically, thus we were motivated to focus on genes that are frequently expressed as measured by snRNA-seq data in order to maximize the mean proportion of non-zero puncta in the nucleus (**Table 1**). Computationally, there are also advantages to focusing on the top 50% expressed genes. Doing so reduces the sparsity of the data, as in snRNA-seq data most genes have 0 count values. See the next answer for a continuation on this point.

**Furthermore, scRNA-seq data is extra-ordinary sparse, where most of genes are unexpressed. Why do not you remove all these unexpressed genes? There is also thresholdd for remove genes with maximum proportion zero (75%).**

As noted by the reviewer, we could have focused on genes that are expressed in all nuclei to resolve sparsity issues and minimize expression rank ties. However, across all 70,527 in the initial snRNA-seq dataset where low quality nuclei had already been removed, no single gene is expressed among all nuclei. There is only one gene expressed in 99.9929% of the nuclei. If we say that 100% is too high and say that we should filter to genes only expressed in 80% or more of the nuclei, we end up with 338 genes (for 75% it would be 527 genes). See https://github.com/LieberInstitute/TREG_paper/blob/0cdf9fbfbd691764a62f9f0fe8d4a0e31d326d 04/code/05_perc_expressed/01_check_for_reviewer.R#L35-L136 for the full breakdown from 0 to 100%. This information is shown as a graph in **Rebuttal Fig. 1**. Using an overall filter like this, one would not take into account that different cell types exist in different proportions, with some cell types much rarer than other ones. This is why we used the maximum proportion zero filter at 75% in order to balance selecting genes that are expressed in most nuclei for all cell types, but don’t absolutely have to be expressed in all nuclei.

**Rebuttal Figure 1: Decaying number of genes expressed in all nuclei.** Y-axis: the number of genes in the 70,522 nuclei in the snRNA-seq data that are expressed in greater or equal to *X*. The X-axis is the percent of nuclei that have expression values (non-zero counts) for a given gene.

While the top 50% and maximum proportion zero at 75% filters are arbitrary, thresholds are needed to deal with the sparsity of the data and reduce ties in expression ranks. Biologically, we also want to select genes observable in most nuclei from all cell types. Filters that balance both the computational requirements as well as the biological goals are needed. We thus built our method with this practical component in mind.

To address this Reviewer’s comment, we have expanded the software documentation (see https://github.com/LieberInstitute/TREG/commit/e6f7f5bebc1e02dfeb5c6a90e28dd760e7025fcf) and added a section on selecting thresholds at

http://research.libd.org/TREG/articles/finding_Total_RNA_Expression_Genes.html#selecting-thr esholds.

**Second, the consistence rank largely depends on the number of cell types, and statistic. However, the authors ignore these two issues. Therefore, the robustness of the proposed method also needs to be addressed.**

In terms of the number of cell types, we completely agree with the reviewer that the candidate TREGs obtained by our method will depend completely on the different cell types in the snRNA-seq dataset used. So even if you use the same snRNA-seq data, but subset to remove a few cell types, the exact rankings of the candidate TREGs will likely be different. But at that point, a user would be re-defining the biological context of interest (i.e. only focused on specific cell types) as the method cannot take into account data it doesn’t see (i.e. dropped cell types). In this sentence in the conclusion, we now highlighted cell types as part of the biological context that TREG depends on:

“As more snRNA-seq data comes online, this Rank Invariance methodology could facilitate identification of TREGs in other experimental settings with differences **in cell types**, donor demographics, brain regions, tissues, or species.”

As for the statistic used, ranks, it is a very general non-parametric statistic that has been used for other methods such as the work we cited by Mar JC et al. and Liu X et al. They used gene expression ranks and consistency among them as part of their normalization methods.

In relation to the robustness of the results, as noted in **Figure 7** and **Table 1**, the slopes for total RNA expression against cell types as well as RNAscope puncta against cell types were comparable once you standardize the units of measurements. For example, -1.33 for snRNA-seq against -1.07 for *AKT3*. So we did use orthogonal data to further investigate our candidate TREGs and consider our method to be robust based on these results.

**2. On the identified TREGs issue, the authors are suggested to exploit the difference between TREGs and non-TREGs from biological perspectives, such as structure, function, etc. Identifying house-keeping genes are insufficient to fully validate the performance of the proposed methods.**

As we have defined a TREG, we have stated that they are context-dependent. So a TREG in the research setting of our interest, human brain cell types in the five regions we studied on neurotypical control donors, could very well have a different biological function than say a TREG in human blood. We are interested in genes that we can quantify in experiments limited to

measuring a small set of genes (i.e. RNAscope experiments where most users probe 4 or less genes) that track with total RNA expression, and thus help us predict total RNA expression from quantifying a single gene. We want these genes to be expressed across heterogenous cell types.

Our own team's initial thought was to use house-keeping genes since they in theory are supposed to be expressed at the same levels across different conditions. However, when we tested many of these housekeeping genes in RNAscope, we observed that they were not expressed in all cell types. This is perhaps not surprising as housekeeping genes were likely identified from bulk tissue studies before single cell technologies were available. That is why we developed the TREG computational method and software– to identify genes (in a data-driven manner) expressed in all cell types whose expression levels track with total RNA amount.

Having found a set of genes useful for our research purposes, we can ask if they are enriched in particular biological functions using GO enrichment analyses. We have updated the GO analysis presented in **Supplementary Figure 5** by adding KEGG enrichment results as well as by checking the top 20 and top 50 candidate TREGs. We added a new **Supplementary Table 2** with the GO and KEGG enrichment results. On the GO results, we noticed that *ARID1B* was frequently among the genes contributing to enriched GO terms whereas *AKT3* contributed to the KEGG enriched pathways.

While the evidence of the biological relevance of these two candidate TREGs as well as others in the top 20 and 50 list is a positive supporting result, we caution against over interpreting these results. That is, while the GO results point towards chromatin binding, histone methylation, and transcription co-regulator/activator activity, which are all related to overall transcription, we caution against expecting candidate TREGs in other biological contexts to be related to these functions. We thus added these changes,

Results:

“Among the genes passing the Proportion Zero filter, the strength of their association with total RNA expression generally increased as their Rank Invariance increased (Supplementary Figure 2b). Furthermore, gene ontology enrichment analysis with the top **20 or 50** candidate TREGs showed that these genes are enriched for key biological processes **such as chromatin binding and transcription regulator activity** (Supplementary Figure 5, Methods: Gene ontology **and KEGG pathway** enrichment analysis). **Among the GO enriched terms, *ARID1B* was a very frequent contributor, whereas for the KEGG enriched pathways, *AKT3* was the principal contributor (Supplementary Table 2**).”

Discussion:

“More generally, other candidate TREGs identified in our neurotypical control tissue are functionally implicated in **chromatin binding and** transcription (Supplementary Figure 5, **Supplementary Table 2**), which is consistent with the notion that a TREG should be predictive of total RNA expression. **However, we do not expect that candidate TREGs in other biological contexts will be enriched for the same biological functions or pathways**.”

Supplementary Figure 5: Gene ontology **and KEGG** dotplots. Gene ontology enrichment results for the top **20 or top 50** candidate TREGs found using snRNA-seq data from 5 human brain regions across ontologies for (A) biological processes (BP), (B) cellular components (CC), (C) molecular function (MF)**, or (D) KEGG pathways**. The top candidate TREGs are enriched for functions related to **chromatin organization, transcription co-regulator/activator activity**, namely RNA splicing and histone modification, as well as related cellular components and biological processes. This finding is consistent with the TREG property that its expression is associated with transcriptional activity. Related to Figure 3c-d.

**3. As methodology, more data are needed to fully validate the performance of the proposed methods.**

We agree with the reviewer that in general more data is useful and appreciated to test methods. However, our existing data demonstrates how the TREG computational method identifies genes that can be quantified with RNAscope that track with total RNA trends seen in snRNA-seq data across heterogenous cell types (**Figure 7**, **Table 1**, **Supplementary Figure 10**).

We are aware that one could aim to further validate a method in new subjects, more brain regions that have different cell types, different tissues that have different cell type populations, cases and controls for different diseases or conditions, among other axes of variation that influence gene expression. We believe that the best way to do this is to share the method with other researchers who have access to diverse data types and the ability to perform validation experiments in these tissues. In this manuscript, we have shown that TREG is successful with data from multiple brain regions and across different cell types. In **Supplementary Figure 11**, we show how data derived from TREG provides important information for heterogeneous cell types that we believe will be useful for resolving challenges in deconvolution, such as the cell size and transcriptional activity factors (see **Figure 1C** of our deconvolution challenges pre-print https://doi.org/10.48550/arXiv.2305.06501).

In the meantime, we have applied the top performing TREG, *AKT3*, in an additional unpublished RNA-scope experiment (n=36; *new RNAscope DLPFC data*) that measures three additional major cell types (Astrocytes, Endothelial cells, and Microglia) in addition to the three cell types we observed in the original submission (Oligodendrocytes, Excitatory Neurons, and Inhibitory Neurons). We have also evaluated the TREG *AKT3* vs. trends of total RNA expression in a recently released human DLPFC snRNA-seq dataset with more samples and nuclei (n = 19, *new snRNA-seq DLPFC data*, Huuki-Myers et al., *bioRxiv*, 2023 doi:

https://doi.org/10.1101/2023.02.15.528722).

Between the snRNA-seq data presented in this manuscript (Tran, Maynard, et al.) and the new snRNA-seq DLPFC data (doi: https://doi.org/10.1101/2023.02.15.528722), we observed a similar pattern of total RNA expression across the three cell types we focused on in this manuscript: Excit > Inhib > Oligo (**Rebuttal Fig. 2A**). In the new RNAscope DLPFC data, we could also replicate the pattern of *AKT3* puncta across the three cell types (**Rebuttal Fig. 2B**). The slopes between the total RNA from the new data were similarly comparable to the original

findings, for snRNA-seq -21844.07 vs. -17093.9 ; for RNAscope -5.52 vs. -6.22 for the TREG and new DLPFC data respectively (**Rebuttal Table 1**).

In this new dataset, we were also able to quantify 3 additional cell types: Endothelial cells, Astrocytes, and Microglia. We were also able to find similar patterns in this expanded biological context with 6 broad cell types in the DLPFC, the 6 cell types are consistent in their order based on medians (**Rebuttal Fig. 2C**&**D**). The new snRNA-seq DLPFC standardized beta is -0.9 versus -1.04 in the new RNAscope DLPFC data for six cell types (**Rebuttal Table 1**).

These analyses show that the patterns observed in the Tran, Maynard, et al. snRNA-seq and TREG RNAscope data replicate in the new snRNA-seq and RNAscope DLPFC datasets. We thus feel confident in the replication of our results across studies.

We have included these data in the rebuttal to address the Reviewer’s concern, but including them in the manuscript is beyond the scope of the present study as they are part of an ongoing project that aims to develop improved deconvolution methods using cell size and activity scale factors.

**4. Finally, as the authors assert that TREGs are highly related to transcriptional activity. Thus, is it possible to generate transcription regulation model with TREGs?**

While our gene ontology enrichment results from the top 20 and top 50 candidate TREGs (**Supplementary Figure 5** and **Supplementary Table 2**) implicate these candidate TREGs in chromatin binding, transcription co-regulator/activator activity among other GO terms related to transcriptional activity and regulation, we don’t necessarily think that candidate TREGs will be related to these functions in other biological contexts. In general, transcriptional regulation has mostly been studied using assays such as ChIP-seq and ATAC-seq, in order to identify where transcription factors bind and where the chromatin is accessible for transcription. Given the rise of multi-omics protocols that measure both snRNA-seq and scATAC-seq (or other omics), one could imagine generating data from a multi-ome assay to further study the relationship between transcription of candidate TREGs derived from the snRNA-seq data and chromatin accessibility control. While it could be interesting to further research this question, we believe that further

exploring this relationship is beyond the scope of this project. In addition, as we have pointed out before, the answer could very well be dependent on the biological context where the candidate TREGs are identified.

**2^nd^ round**

**Reviewer 1**

Huuki-Myers et al. have answered all my questions. I recommend that we accept their manuscript.

**Reviewer 2**

The authors properly address all my comments. Now, I have no further questions.
